# Ursodeoxycholic acid does not reduce SARS-CoV-2 infection in newly allogeneic hematopoietic stem cell transplantation recipients: a prospective NICHE cohort

**DOI:** 10.3389/fcimb.2024.1324019

**Published:** 2024-03-05

**Authors:** Hongye Gao, Jiali Wang, Xinhui Zheng, Xiaolei Pei, Yawei Zheng, Weihua Zhai, Rongli Zhang, Xin Chen, Qiaoling Ma, Jialin Wei, Donglin Yang, Aiming Pang, Yi He, Sizhou Feng, Yigeng Cao, Erlie Jiang

**Affiliations:** ^1^ State Key Laboratory of Experimental Hematology, National Clinical Research Center for Blood Diseases, Haihe Laboratory of Cell Ecosystem, Institute of Hematology & Blood Diseases Hospital, Chinese Academy of Medical Sciences & Peking Union Medical College, Tianjin, China; ^2^ Tianjin Institutes of Health Science, Tianjin, China

**Keywords:** Ursodeoxycholic acid, hematopoietic stem cell transplantation, SARS-CoV-2, prevention and control, cohort studies

## Abstract

**Introduction:**

Retrospective studies have suggested that Ursodeoxycholic Acid (UDCA) provide a protective effect against SARS-CoV-2 infection, particularly in patients with liver disease. However, it is uncertain whether this finding can be extended to the allogeneic hematopoietic stem cell transplantation (allo-HSCT) cohort. Therefore, we aim to examine the protective potential of UDCA against SARS-CoV-2 infection in recently received allo-HSCT patients.

**Methods:**

During the initial Omicron variant wave in China (December 2022 to February 2023), we conducted a prospective observational study involving 91 hospitalized patients who had undergone allo-HSCT within the previous 6 months as part of the National Longitudinal Cohort of Hematological Diseases (NICHE). Throughout hospitalization, we continuously monitored the status of COVID-19 using SARS-CoV-2 PCR kits or SARS-CoV-2 Antigen Rapid Tests.

**Results:**

Among these patients, 67.0% (n = 61) were confirmed to have contracted SARS-CoV-2 infection. For the 52 patients evaluated, 23.1% experienced a severe or critical clinical course. There was no difference in the infection rate or severity of COVID-19 between the UDCA group and the non-UDCA group. We found that only patients transplanted between 3 and 6 months ago demonstrated a higher risk of SARS-CoV-2 infection compared to those who received allo-HSCT within 3 months (Odds Ratio [OR]: 3.241, 95% Confidence Interval [CI]: 1.287-8.814, *P* = 0.016). But other clinical factors, such as administration of UDCA, showed no difference. Notably, only age ≥38 years old remained as an independent risk factor for a severe clinical course of SARS-CoV-2 infection (OR: 3.664, 95% CI: 1.129-13.007, *P* = 0.035).

**Conclusion:**

The effectiveness of UDCA in protecting newly allo-HSCT recipients against SARS-CoV-2 infection remains unconfirmed. Presently, the most effective strategy appears to be minimizing exposure to SARS-CoV-2.

**Clinical trial registration:**

https://clinicaltrials.gov/ct2/show/NCT04645199, identifier NCT04645199.

## 1 Introduction

Allogeneic hematopoietic stem cell transplantation (allo-HSCT) is a significant curative strategy primarily utilized for the treatment of hematological disorders (Khaddour et al., 2023). This therapy entails the reconstruction of the recipient’s hematopoietic and immune systems, a process that can potentially render them more vulnerable to infections caused by various pathogens (Annaloro et al., 2020; Gilis et al., 2014). Notably, the novel severe acute respiratory syndrome coronavirus-2 (SARS-CoV-2) is the etiologic agent of Coronavirus disease 19 (COVID-19), occasionally giving rise to outbreaks with variant strains (Bordat et al., 2023). In the setting of human beings of long-term coexistence with SARS-CoV-2, allo-HSCT recipients with rejuvenated immune systems are a subject of heightened concern.

Considerable endeavors have been made to mitigate SARS-CoV-2 infection in allo-HSCT recipients. However, immunogenicity of SARS-CoV-2 vaccination of two-dose vaccination strategies proved insufficient after transplant (Murray et al., 2022). Administering a new vaccine dose to these recipients may fail to establish immune response for their deficient immune systems (Einarsdottir et al., 2022), and even pose a risk of exacerbating graft-versus-host disease (GVHD) (Massoud et al., 2022). In addition, convalescent plasma therapy may lose its effectiveness for mutations in the viral strain (Cao et al., 2022). There is still a long way to go in effectively preventing SARS-CoV-2 infection in this vulnerable population.

One promising strategy is the downregulation of host receptors for SARS-CoV-2. Angiotensin-Converting Enzyme 2 (ACE2) serves as the primary receptor, and nearly the exclusive one, for the entry of SARS-CoV-2 into human cells (Tan et al., 2020). Recently, Brevini et al. have presented compelling evidence that Ursodeoxycholic acid (UDCA) can effectively downregulate ACE2 expression by reducing farnesoid X receptor signaling (Brevini et al., 2023). This mechanism holds the potential to reduce susceptibility to SARS-CoV-2 infection, supporting the idea that UDCA could have a positive impact on COVID-19 outcomes, especially in patient with liver disease (John et al., 2023). Currently, emerging clinical evidence suggests that utilizing UDCA as a pharmacological prophylaxis against SARS-CoV-2 infection in vulnerable groups. But prospective and robust clinical evidence are lacking. Additionally, the protective effects of UDCA in preventing SARS-CoV-2 infection remain controversial, especially in immunocompromised group (Colapietro et al., 2023).

While UDCA has been extensively used for prophylaxis against sinusoidal obstruction syndrome following allo-HSCT in clinical practice (Essell et al., 1992), the efficacy of UDCA to reduce susceptibility to SARS-CoV-2 infection in this population remains uninvestigated. Therefore, we aim to identify clinical factors associated with SARS-CoV-2 infection among recipients of allo-HSCT, and assess the potential impact of UDCA on the susceptibility of this population to COVID-19. This research will be carried out within the prospective National Longitudinal Cohort of Hematological Diseases (NICHE), registered under NCT04645199, during initial period of the Omicron variant wave in China of 2022.

## 2 Materials and methods

### 2.1 Participants and treatments

We conducted a prospective data collection study involving patients who underwent allo-HSCT within a 6-month period in the NICHE project (NCT04645199). Data was collected from December 2022 to February, 2023.

All patients received myeloablative conditioning regimen. Patients with an unrelated or mismatched related donor received rabbit anti-thymocyte globulin (ATG) at 2 to 2.5 mg/kg/day (days -4 to -1). Each patient underwent GVHD prophylaxis according to protocol: cyclosporine A (1 mg/kg/d, starting from day -5) or tacrolimus (0.03 mg/kg/d, starting from day -1), in conjunction with short-course methotrexate (MTX, 15 mg/m^2^ on day +1; 10 mg/m^2^ on days +3, +6, and +11), with or without mycophenolate mofetil (1,000 mg/d). Glucocorticoids were employed as first-line treatment for GVHD, while second-line treatment options included CD25 monoclonal antibodies, mesenchymal stem cells (MSCs), or ruxolitinib.

UDCA was administered as a precautionary routine following allo-HSCT, with a standardized dosage of 250 mg taken orally three times daily in our center. However, in clinical practice, some patients deviated from the prescribed UDCA administration for intestinal GVHD, clinical considerations, or personal reasons. In terms of the administration of UDCA, we classified enrolled patients into two groups: the UDCA-treated group (UDCA group) and the UDCA-untreated group (non-UDCA group). Participants in the UDCA group were required to take UDCA for clinical care for a minimum of 2 weeks before enrollment. Throughout the study period, patients in the UDCA group continued to receive UDCA at a fixed dose of 250 mg three times a day, orally, at least until the end of the observation period. Patients who halted UDCA treatment for more than 4 weeks before confirming a positive test result were categorized as part of the non-UDCA group, as referenced in Brevini’s study (Brevini et al., 2023).

Neutrophil engraftment was characterized by achieving an absolute neutrophil count of ≥0.5 × 10^9^/L for three consecutive days in peripheral blood without the use of growth factor support. Platelet engraftment was defined as maintaining an untransfused platelet count of ≥20 × 10^9^/L for seven consecutive days.

### 2.2 COVID-19 assessment

SARS-CoV-2 infection was continually monitored through the utilization of both a SARS-CoV-2 PCR kit and a SARS-CoV-2 Antigen Rapid Test. The severity of COVID-19 was evaluated according to the criteria outlined in the COVID-19 Treatment Guidelines established by the US National Institutes of Health (COVID-19 Treatment Guidelines Panel). We also evaluated plasm angiotensin-converting enzyme 2 (ACE2) activity by kit according to the manufacturer’s instructions (Beyotime, Jiangsu, China; Cat. P0319S).

### 2.3 Statistical analysis

Logistic regression analyses were performed to determine risk factors for SARS-CoV-2 infection, with significant factors (*P* < 0.05) from univariate analysis subjected to multivariate analysis. Statistical analysis was performed using R software version 4.2.0 (https://www.r-project.org/about.html). For categorical variables, percentages were used to summarize the data and tested using Fisher’s exact test or the chi-squared test. Normality was assessed using the Shapiro-Wilk test. For continuous variables, the non-parametric Mann-Whitney test or the two-tailed Student’s t-test was used to compare data between two groups, depending on the data distribution. One-way analyses of variance (ANOVA) were used to compare the means of multiple groups. We considered a *P* value of less than 0.05 to be statistically significant.

## 3 Results

### 3.1 Overview of SARS-CoV-2 infection

The median age of the enrolled patients was 38 years, with a male-to-female ratio of 1:1.6. More than half of the patients underwent hematopoietic stem cell transplantation, as detailed in **Table 1**. The median time to transplant was 83 days (range: 9-183 days). The clinical parameters are comparable between the UDCA group and the non-UDCA group (**Table 1**; **Supplementary Table S1**).

**Table 1:** Patients’ baseline.

Characteristic	Overall	Non-UDCA	UDCA	*P* Value
**Number of patients**	91	13	78	
Age				
Median (IQR)	38.0 (29.5, 47.0)	31.0 (22.0, 42.0)	38.0 (30.3, 48.0)	0.116
Age ≥ 38, *n* (%)	46 (50.5)	6 (46.2)	40 (51.3)	0.966
**Male, *n* (%)**	56 (61.5)	8 (61.5)	48 (61.5)	> 0.999
**CCI score ≥ 2, *n* (%)**	17 (18.7)	2 (15.4)	15 (19.2)	> 0.999
**Diabetes Mellitus, *n* (%)**	12 (13.2)	1 (7.7)	11 (14.1)	0.850
**ECOG score (%)**				0.100
0	28 (30.8)	5 (38.5)	23 (29.5)	
1	37 (40.7)	8 (61.5)	29 (37.2)	
2	19 (20.9)	0 (0.0)	19 (24.4)	
3	7 (7.7)	0 (0.0)	7 (9.0)	
**Disease, *n* (%)**				0.281
ALL	25 (27.5)	2 (15.4)	23 (29.5)	
AML	34 (37.4)	8 (61.5)	26 (33.3)	
MDS	12 (13.2)	1 (7.7)	11 (14.1)	
Others^*^	20 (22.0)	2 (15.4)	18 (23.1)	
**Disease status before transplant, *n* (%)**				0.156
Early/low	62 (68.1)	6 (46.2)	56 (71.8)	
Intermediate	23 (25.3)	6 (46.2)	17 (21.8)	
High/advanced	6 (6.6)	1 (7.7)	5 (6.4)	
**Donor-recipient sex match, *n* (%)**				
Female to Male	17 (18.7)	0 (0.0)	17 (21.8)	0.138
**Transplant types, *n* (%)**				0.577
HID	54 (59.3)	6 (46.2)	48 (61.5)	
MSD	32 (35.2)	6 (46.2)	26 (33.3)	
URD	5 (5.5)	1 (7.7)	4 (5.1)	
**Auxiliary UCBT, *n* (%)**	9 (9.9)	1 (7.7)	8 (10.3)	> 0.999
Engraftment post transplantation, *n* (%)				
Neutrophil	87 (95.6)	13 (100.0)	74 (94.9)	0.917
Platelet	80 (87.9)	12 (92.3)	68 (87.2)	0.948
**Acute GVHD, *n* (%)**	28 (30.8)	3 (23.1)	25 (32.1)	0.746
Time after transplantation				
≥ 3 months, n (%)	41 (45.1)	8 (61.5)	33 (42.3)	0.323

ECOG, Eastern Cooperative Oncology Group performance status; CCI, Charlson Comorbidity Index (excluding primary disease); ALL, Acute lymphoblastic leukemia; AML, Acute myeloid leukemia; MDS, Myelodysplastic syndrome; UCBT, Umbilical cord blood transplantation; HID, haploidentical donor; MSD, HLA-matched sibling donor; URD, Unrelated donor; UDCA, Ursodeoxycholic acid; GVHD, Graft-versus-host-disease.

*Others indicate patients with lymphomas, multiple myeloma, aplastic anemia, chronic myelomonocytic leukemia, and mixed phenotype acute leukemia.

During the observation period, SARS-CoV-2 infection was identified in 67.0% (n = 61) of the patients. Among the 52 patients for whom evaluation was possible, the distribution of symptom severity was as follows: asymptomatic/mild in 51.9%, moderate in 25.0%, and severe/critical in 23.1%. Thirty-two patients exhibited abnormal chest imaging associated with SARS-CoV-2. The most frequently reported clinical symptoms associated with SARS-CoV-2 infection were cough (89.1%), fatigue (77.4%), pharyngalgia (57.4%), myalgia (50.0%), gustatory dysfunctions (47.1%). We noted comparable COVID-19-related symptoms and severity between the UDCA group and the non-UDCA group (**Table 2**). By the last follow-up date, 2 patients had succumbed to complications arising from SARS-CoV-2 infection. The mean time from confirmation of SARS-CoV-2 infection to decease was 9.5 days.

**Table 2:** COVID-19-related symptoms, severity and mortality.

COVID-19 symptoms	Overall	Non-UDCA	UDCA	*P* value
**Number of patients**	61	9	52	
**Fever, *n* (%)**	40 (70.2)	5 (55.6)	35 (72.9)	0.517
Tmax °C, median (IQR)^a^	38.4 (38.1, 38.9)	38.5 (38.4, 38.7)	38.4 (38.0, 38.9)	0.555
Frequency, median (IQR)^b^	1.0 (1.0, 2.0)	1.5 (1.0, 2.0)	1.0 (1.0, 2.5)	0.443
Days of fever with Tmax, median (IQR)	2.0 (1.0, 3.8)	2.0 (1.0, 3.0)	2.0 (1.0, 4.0)	0.746
**Hypoxaemia, *n* (%)**	16 (29.6)	3 (33.3)	13 (28.9)	> 0.999
**Abdominal pain, *n* (%)**	12 (21.8)	3 (33.3)	9 (19.6)	0.636
**Cough, *n* (%)**	49 (89.1)	8 (88.9)	41 (89.1)	> 0.999
**Polypnea, *n* (%)**	23 (42.6)	4 (44.4)	19 (42.2)	> 0.999
**Gustatory dysfunctions, *n* (%)**	24 (47.1)	5 (62.5)	19 (44.2)	0.571
**Diarrhea, *n* (%)**	15 (27.3)	2 (22.2)	13 (28.3)	> 0.999
**Myalgia, *n* (%)**	27 (50.0)	4 (44.4)	23 (51.1)	> 0.999
**Fatigue, *n* (%)**	41 (77.4)	7 (77.8)	34 (77.3)	> 0.999
**Pharyngalgia, *n* (%)**	31 (57.4)	4 (44.4)	27 (60.0)	0.623
**Aggravating GVHD reported, *n* (%)**	6 (30.0)	0 (0.0)	6 (42.9)	0.166
**Severity of COVID-19, *n* (%)**				0.517
Asymptomatic	1 (1.9)	0 (0.0)	1 (2.3)	
Mild	26 (50.0)	5 (62.5)	21 (47.7)	
Moderate	13 (25.0)	3 (37.5)	10 (22.7)	
Critical	2 (3.8)	0 (0.0)	2 (4.5)	
Severe	10 (19.2)	0 (0.0)	10 (22.7)	
**Prescription of Paxlovid/Azvudine, *n* (%)**	19 (31.1)	2 (22.2)	17 (32.7)	0.813
**PCR-negative conversion, days, median (IQR)**	15.0 (10.0, 27.0)	19.0 (10.0, 25.0)	15.0 (9.8, 27.3)	0.842
**ICU admission, *n* (%)**	2 (3.3)	0 (0.0)	2 (3.8)	> 0.999
**COVID-19-related mortality, *n* (%)**	2 (3.3)	0 (0.0)	2 (3.8)	> 0.999

a Tmax, maximum temperatures.

b Daily number of fever peaks.

### 3.2 Clinical risk factors for SARS-CoV-2 infection

We observed that patients with a time to transplantation of ≥3 months had a higher risk of infection compared to those transplanted between 3 and 6 months (OR: 3.241, 95% CI: 1.287-8.814, *P* = 0.016). However, other potential factors, including UDCA prophylaxis, did not reach statistical significance in the univariate analysis (**Table 3**). To further mitigate confounding, we performed propensity score matching (1:1) to control for age, gender, transplant type, and primary disease among individuals who received UDCA (n = 13) and their matched counterparts without UDCA (n = 13, data not shown). Our analysis revealed no significant difference in the SARS-CoV-2 infection rate between the two matched cohorts (76.9% vs. 69.2%, *P* > 0.999).

**Table 3:** Univariate analysis for the clinical factors of SARS-CoV-2 infection.

Characteristics	OR	95%CI	*P* value
**Age ≥ 38, *n* (%)**	2.325	0.959 to 5.858	0.066
**Male, *n* (%)**	0.72	0.281 to 1.773	0.481
**CCI score ≥ 2, *n* (%)**	0.644	0.219 to 1.971	0.427
**ECOG ≥ 2, *n* (%)**	1.151	0.441 to 3.185	0.778
**UDCA prophylaxis, *n* (%)**	0.889	0.224 to 3.013	0.856
Disease, *n* (%)			
ALL	Ref.	Ref.	–
AML	1.129	0.362 to 3.465	0.831
MDS	0.659	0.159 to 2.732	0.565
Other	0.874	0.252 to 3.036	0.832
Disease status before transplant, *n* (%)			
Early/low	Ref.	Ref.	–
Intermediate	0.938	0.14 to 6.28	0.947
High/Advanced	1.05	0.177 to 6.22	0.957
**Auxiliary UCBT, *n* (%)**	1.815	0.448 to 7.353	0.475
Donor-recipient sex match, *n* (%)			
Female-Male	1.224	0.406 to 12.727	0.730
Transplant types, *n* (%)			
MSD	Ref.	Ref.	–
HID	0.614	0.231 to 1.629	0.327
URD	0.222	0.031 to 1.578	0.133
Engraftment post transplantation, *n* (%)			
Neutrophil	2.107	0.243 to 18.308	0.468
Platelet	1.187	0.29 to 4.299	0.798
**Acute GVHD, *n* (%)**	1.056	0.414 to 2.815	0.911
Time after transplantation, *n* (%)			
≥ 3 months	3.241	1.287 to 8.814	**0.016**

OR, Odds Ratio; CI, Confidence Interval.

Significant difference (P < 0.05) is bolded.

### 3.3 Clinical risk factors for severe clinical course

We categorized patients with confirmed SARS-CoV-2 infection into two groups: those with a mild clinical course (asymptomatic/mild) and those with a severe clinical course (moderate/severe/critical). Among the clinical factors mentioned above, age≥38 years (OR: 3.740, 95% CI: 1.206-12.572, *P* = 0.026) and Eastern Cooperative Oncology Group performance status (ECOG) score ≥2 (OR: 3.833, 95% CI: 1.071-16.113, *P* = 0.048) demonstrated significant associations with a severe clinical course in the univariate analysis. And the multivariate analysis revealed that only age ≥38 years was independent risk factor for the severe clinical course (OR: 3.664, 95%CI: 1.129-13.007, *P* = 0.035). Other clinical parameters, including UDCA prophylaxis, transplant types, and acute GVHD status, did not provide significant insights into the severity of COVID-19 (**Table 4**).

**Table 4:** Univariate analysis for the clinical factors of severity of COVID-19.

Characteristics	OR	95%CI	*P* value
**Age ≥ 38, *n* (%)**	3.740	1.206 to 12.572	**0.026**
**Male, *n* (%)**	1.018	0.339 to 3.071	0.974
**CCI score ≥ 2, *n* (%)**	4.861	1.032 to 35.283	0.066
**ECOG ≥ 2, *n* (%)**	3.833	1.071 to 16.113	**0.048**
**UDCA prophylaxis, *n* (%)**	1.667	0.364 to 8.936	0.518
Disease, *n* (%)			
ALL	Ref.	Ref.	–
AML	1.227	0.316 to 4.921	0.767
MDS	6.000	0.677 to 134.0	0.147
Others	1.500	0.323 to 7.197	0.604
Disease status before transplant, *n* (%)			
Early/low	Ref.	Ref.	–
Intermediate	4	0.273 to 58.565	0.311
High/Advanced	1.524	0.127 to 18.325	0.740
**Auxiliary UCBT, *n* (%)**	1.52	0.303 to 8.486	0.608
Donor-recipient sex match, *n* (%)			
Female-Male	1.100	0.269 to 4.506	0.892
Transplant types, *n* (%)			
MSD	Ref.	Ref.	–
HID	2.082	0.657 to 6.973	0.219
URD	1.714	0.061 to 48.174	0.718
Engraftment post transplantation, *n* (%)			
Neutrophil	–	–	–
Platelet	0.917	0.155 to 5.41	0.920
**Acute GVHD, *n* (%)**	2.475	0.715 to 9.409	0.162
Time after transplantation, *n* (%)			
≥ 3 months	1.651	0.547 to 5.139	0.377

Significant differences (P < 0.05) are bolded.

Additionally, we conducted an exploratory *post hoc* subgroup analysis. No significant differences were observed in the rate of SARS-CoV-2 infection or the severity of the disease among subgroups of patients receiving UDCA treatment compared to those not receiving UDCA (**Supplementary Figure S1**).

### 3.4 Plasma ACE2 activity in allo-HSCT recipients

It is well-known that SARS-CoV-2 gains entry into host cells through ACE2 receptors and downregulates ACE2 expression within cells, but its effects on soluble ACE2 levels remain contradictory (García-Ayllón et al., 2021; Nagy et al., 2021; van Lier et al., 2021; Daniell et al., 2022). We furthermore assessed the plasma relative ACE2 activity in patients receiving UDCA and those who did not. However, there were no between-groups differences as expected (**Supplementary Figure S2A**).

Then, we incorporated a recovery cohort (n = 13) and assessed their relative ACE2 activity following the day of PCR-negative conversion. We found no significant changes in the relative ACE2 activity levels between individuals who were administered UDCA and those who were not, as observed at three different time points (day 14, 28, and 56; see **Supplementary Figure S2B**). These findings collectively suggest that UDCA had a limited impact on plasma ACE2 activity.

## 4 Discussion

This study represents the first prospective investigation into the clinical outcomes of SARS-CoV-2 infection among allo-HSCT recipients during the initial Omicron wave of 2022 in China. Importantly, in contrast to prior positive findings regarding the potential of UDCA to prevent SARS-CoV-2 infection, our research reveals that the use of UDCA does not provide any preventive or mitigating effects against SARS-CoV-2 infection within this specific population. The search for more effective preventive measures in immunocompromised patients remains ongoing.

Preclinical and some retrospective studies support the prospective effects of UDCA on SARS-CoV-2 infection. Brevini et al. demonstrated that ACE2 downregulation mediated by UDCA reduced SARS-CoV-2 infection *in vivo* and *in vitro*, providing evidence that SARS-CoV-2 infection could be reduced using UDCA (Brevini et al., 2023). And some investigators conducted retrospective studies of cohorts comprising UDCA-treated patients with liver disease, support the UDCA efficacy on SARS-CoV-2 infection (Brevini et al., 2023; John et al., 2023; Li et al., 2023). However, some researchers have demonstrated that UDCA did not decrease susceptibility to SARS-CoV-2 infection, in both children (Liu and Wang, 2023) and hospitalized patients (Colapietro et al., 2023), consistent with the findings of the present study.

The differing interpretations of previous study results can be attributed to several factors. Firstly, variations in patient demographics may lead to diverse outcomes. In our study, we concentrated on allo-HSCT patients, whose immune systems were compromised by the transplant procedure. These patients required long-term immunosuppressive therapy to prevent allograft rejection, making them more vulnerable to infections (Annaloro et al., 2020; Gilis et al., 2014).

In contrast, Li’s and John’s studies focused on patients with chronic liver diseases, including cholestasis and cirrhosis (John et al., 2023; Li et al., 2023). This population may have compromised immune systems due to the underlying condition itself, such as chronic viral infections or autoimmune-mediated liver damage (Heymann and Tacke, 2016). However, they may not necessarily be subject to continuous immunosuppressive medication as in the case of allo-HSCT procedure. Despite potentially varying degrees of compromised immune function, these patients still retain some capacity to mount an immune response to SARS-CoV-2, particularly among those who have been vaccinated (Marjot et al., 2021; Ozaka et al., 2022).

Most importantly, the dosage regimen merits consideration. In our study, administered a daily UDCA dosage of 750mg, exceeding the standard recommendation (Van Hoogstraten et al., 1998), which was lower than or equal to the dose range (750-1, 250mg/day) used in the study conducted by Brevini et al. (Brevini et al., 2023). The variation in dosage levels may account for the inconsistency in the effectiveness observed in our study. Furthermore, a portion of the research relies on self-reported COVID-19 diagnoses by patients, which may lead to bias, whereas our study utilizes hospital-reported testing as the diagnostic standard. Overall, there still remains a high infection rate of SARS-CoV-2 despite the majority of our study participants receiving UDCA, which dims the prospects for UDCA in preventing SARS-CoV-2 infection.

The current clinical evidence focus on UDCA impact on SARS-CoV-2 infection was summarized in **Supplementary Table S2**. While some cohorts have indicated a protective effect of UDCA against SARS-CoV-2 infection (Webb et al., 2020; Marjot et al., 2021; Rogiers et al., 2021; Bordat et al., 2023; Brevini et al., 2023), this effect seems to be less pronounced in allo-HSCT recipients. Part of the inconsistency may stem from the diverse biological characteristics present within our study population. However, our study was hampered by the limitation of a small sample size, highlighting the need for more extensive investigations in this issue.

Based on results of logistic regression, only the time elapsed after allo-HSCT (>3 months *vs*. <3 months) was associated with SARS-CoV-2 infection. Typically, patients who have surpassed the 3-month mark after transplantation are more likely to be discharged from hospital surveillance and may have increased contact with infected individuals. However, the time after transplant did not show a significant association with the severity of the clinical course. Consistent with findings in other reports concerning vulnerable populations, older age (Webb et al., 2020; Sharma et al., 2021) and a poorer ECOG status (Grivas et al., 2021; Rogiers et al., 2021) were associated with a higher severity of COVID-19.

The protective effects of UDCA against SARS-CoV-2 infection were not substantiated in newly allo-HSCT recipients. Reducing exposure to SARS-CoV-2 is currently the most effective strategy, while improved prevention strategies are in development.

## Data availability statement

The raw data supporting the conclusions of this article will be made available by the authors, without undue reservation.

## Ethics statement

This project received ethical approval from the Institute of Hematology & Blood Diseases Ethics Committee (IIT2021011-EC-1). The studies were conducted in accordance with the local legislation and institutional requirements. The participants provided their written informed consent to participate in this study.

## Author contributions

HG: Funding acquisition, Formal analysis, Methodology, Writing – original draft. JWa: Formal analysis, Methodology, Writing – review & editing. XZ: Methodology, Writing – review & editing, Data curation. XP: Writing – review & editing, Investigation, Supervision, Visualization. YZ: Supervision, Writing – review & editing. WZ: Supervision, Writing – review & editing. RZ: Supervision, Writing – review & editing. XC: Supervision, Writing – review & editing. QM: Supervision, Writing – review & editing. JWe: Supervision, Writing – review & editing. DY: Supervision, Writing – review & editing. AP: Supervision, Writing – review & editing. YH: Supervision, Writing – review & editing. SF: Supervision, Writing – review & editing. YC: Supervision, Writing – review & editing. EJ: Supervision, Writing – review & editing, Conceptualization, Funding acquisition, Resources.
