# Supplementary material for: Ursodeoxycholic acid does not reduce SARS-CoV-2 infection in newly allogeneic hematopoietic stem cell transplantation recipients: a prospective NICHE cohort
Source: Front Cell Infect Microbiol. 2024 Mar 5;14:1324019. doi: 10.3389/fcimb.2024.1324019 (PMC10949982; doi:10.3389/fcimb.2024.1324019)
Supplement: Supplementary file 1 [file DataSheet_1.pdf]

Supplementary Material

Supplementary Figure 1

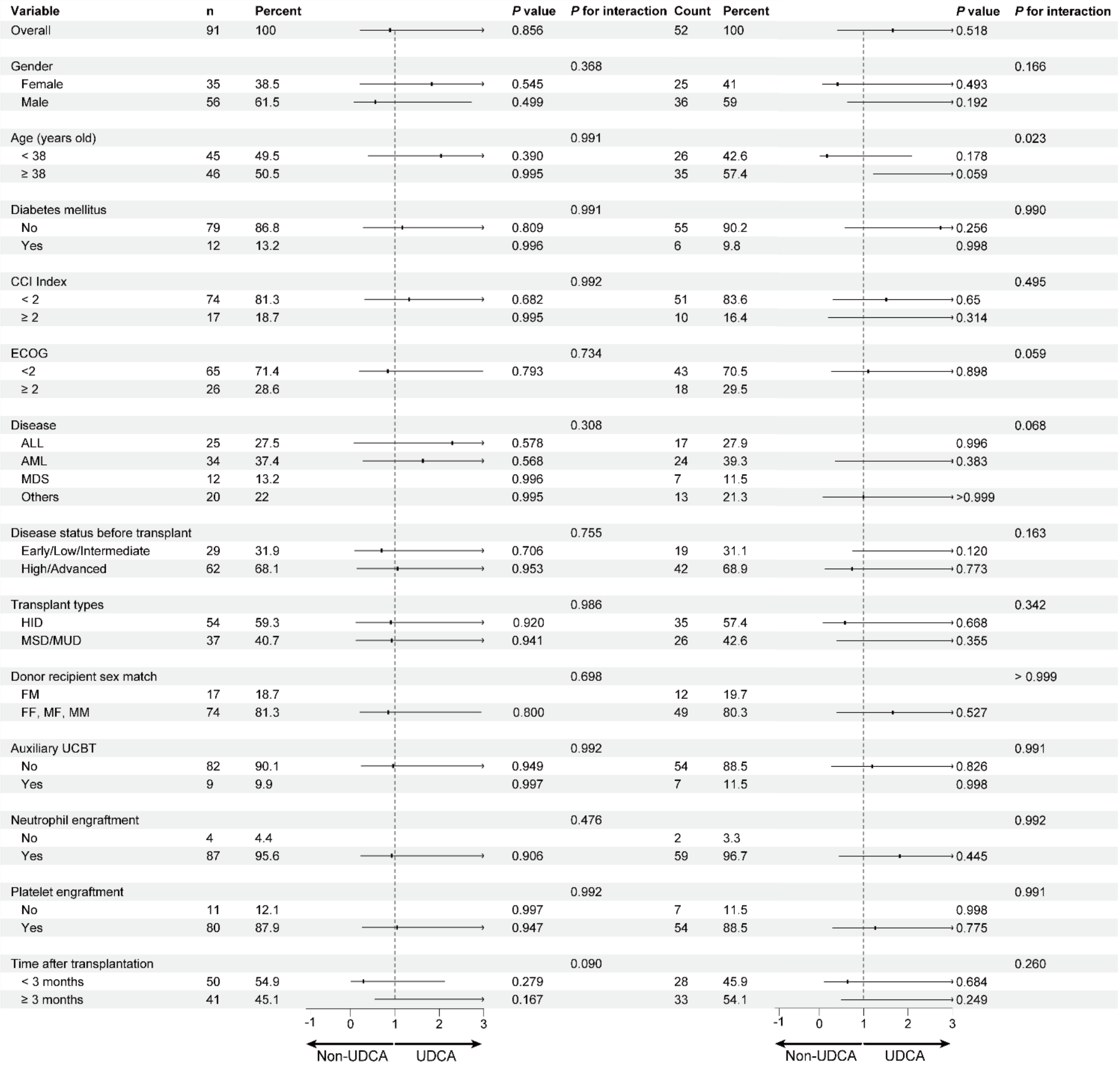

Variable	n	Percent	*P* value	*P* for interaction	Count	Percent	*P* value	*P* for interaction
Overall	91	100	0.856		52	100	0.518	
Gender				0.368				0.166
Female	35	38.5	0.545		25	41	0.493	
Male	56	61.5	0.499		36	59	0.192	
Age (years old)				0.991				0.023
< 38	45	49.5	0.390		26	42.6	0.178	
≥ 38	46	50.5	0.995		35	57.4	0.059	
Diabetes mellitus				0.991				0.990
No	79	86.8	0.809		55	90.2	0.256	
Yes	12	13.2	0.996		6	9.8	0.998	
CCI Index				0.992				0.495
< 2	74	81.3	0.682		51	83.6	0.65	
≥ 2	17	18.7	0.995		10	16.4	0.314	
ECOG				0.734				0.059
<2	65	71.4	0.793		43	70.5	0.898	
≥ 2	26	28.6			18	29.5		
Disease				0.308				0.068
ALL	25	27.5	0.578		17	27.9	0.996	
AML	34	37.4	0.568		24	39.3	0.383	
MDS	12	13.2	0.996		7	11.5		
Others	20	22	0.995		13	21.3	>0.999	
Disease status before transplant				0.755				0.163
Early/Low/Intermediate	29	31.9	0.706		19	31.1	0.120	
High/Advanced	62	68.1	0.953		42	68.9	0.773	
Transplant types				0.986				0.342
HID	54	59.3	0.920		35	57.4	0.668	
MSD/MUD	37	40.7	0.941		26	42.6	0.355	
Donor recipient sex match				0.698				> 0.999
FM	17	18.7			12	19.7		
FF, MF, MM	74	81.3	0.800		49	80.3	0.527	
Auxiliary UCBT				0.992				0.991
No	82	90.1	0.949		54	88.5	0.826	
Yes	9	9.9	0.997		7	11.5	0.998	
Neutrophil engraftment				0.476				0.992
No	4	4.4			2	3.3		
Yes	87	95.6	0.906		59	96.7	0.445	
Platelet engraftment				0.992				0.991
No	11	12.1	0.997		7	11.5	0.998	
Yes	80	87.9	0.947		54	88.5	0.775	
Time after transplantation				0.090				0.260
< 3 months	50	54.9	0.279		28	45.9	0.684	
≥ 3 months	41	45.1	0.167		33	54.1	0.249	

Supplementary Figure 1. In an exploratory post hoc subgroup analysis, it was found that there were no significant differences in either the rate (left) or severity (right) of SARS-CoV-2 infection among most subgroups of patients who received UDCA treatment compared to those who did not. However, the number of cases in certain subgroups (age >38 years old, diabetes mellitus, and others) within the non-UDCA group was insufficient to conduct statistically meaningful analysis.

ECOG: Eastern Cooperative Oncology Group performance status; **CCI:** Charlson Comorbidity Index (excluding primary disease); **ALL:** Acute lymphoblastic leukemia; **AML:** Acute myeloid leukemia; **MDS:** Myelodysplastic syndrome; **UCBT:** Umbilical cord blood transplantation; **HID:** haploidentical donor; **MSD:** HLA-matched sibling donor**; URD:** unrelated donor**; UDCA:** Ursodeoxycholic acid; **GVHD:** Graft-versus-host-disease

* Others indicate patients with lymphomas, multiple myeloma, aplastic anemia, chronic myelomonocytic leukemia, and mixed phenotype acute leukemia.

Supplementary Figure 2

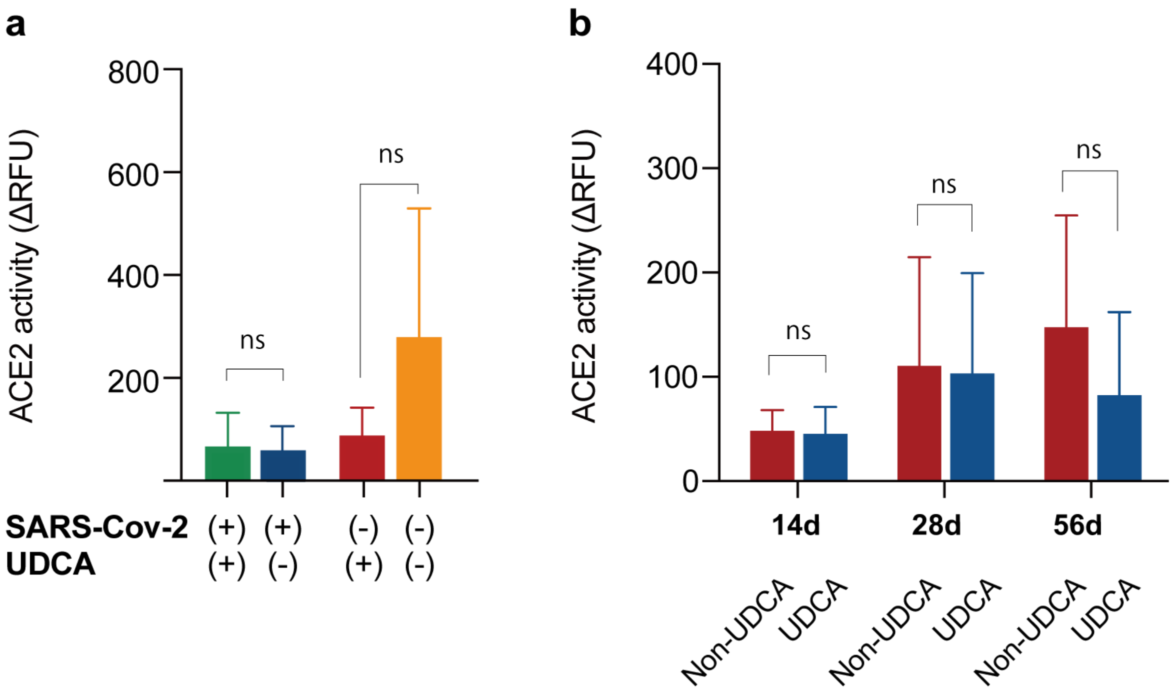

Supplementary Figure 2. Evaluation of ACE2 activity (RFU/min) in allo-HSCT recipients (a). Patients were divided into four groups based on SARS-CoV-2 infection status and UDCA administration: SARS-CoV-2 test positive with UDCA taken (green) or without UDCA taken (blue); SARS-CoV-2 test negative patients with UDCA (red) or without UDCA (yellow). The changes in ACE2 RFU are shown in for recovery patients with SARS-CoV-2 infection. Samples were assessed at 14, 28, and 56 days since the date of PCR-negative conversion (b). Error bars indicate means + SD. $^*P < 0.05$; ns: nonsignificant.

RFU: Relative fluorescence units

Supplementary Table 1. Baseline laboratory hematological indicators

	**Overall**	**Non-UDCA**	**UDCA**	***P* value**
**Number of patients**	91	13	78	
**Absolute values (mean ± SD) × 10^9/L**				
White blood cells	3.55 ± 1.92	3.38 ± 1.01	3.58 ± 2.04	0.733
Lymphocytes	0.87 ± 0.90	1.26 ± 0.60	0.80 ± 0.93	0.093
Neutropenia	2.13 ± 1.38	1.68 ± 0.69	2.21 ± 1.46	0.205
Platelets	82.63 ± 60.11	101.67 ± 50.75	79.71 ± 61.18	0.241
**Abnormal liver function, *n* (%)**				
ALT and/or AST levels were higher than normal	69 (75.8)	10 (76.9)	59 (75.6)	> 0.999

ALT: alanine aminotransferase**; AST:** aspartate aminotransferase

Supplementary Table 2. Clinical reports of impact of UDCA against SARS-CoV-2 infection

Publication title	Authors	Disease	Cohort	UDCA (n)	Non-UDCA (n)	Protective effect of UDCA on SARS-CoV-2 infection	COVID-19 clinical outcome
FXR inhibition may protect from SARS-CoV-2 infection by reducing ACE2	Brevini, *et al.*(1)	Chronic liver disease	COVID-Hep/SECURE17 Liver registries	31	155	Yes	UDCA treatment led to better outcomes with reduced hospitalization, ICU admission, and mortality compared to no UDCA treatment.
FXR inhibition may protect from SARS-CoV-2 infection by reducing ACE2	Brevini, *et al.*(1)	Liver transplant recipients who received at least two doses of a COVID-5 19 mRNA vaccine	Costs Associated with Liver disease (VOCAL) cohort	24	72	Yes	UDCA users had a significantly lower risk of developing moderate, severe, or critical COVID-19
Protective effect of ursodeoxycholic acid on COVID-19 in patients with chronic liver	Li, *et al.* (2)	Chronic liver disease	NA	225	225	Yes	UDCA reduces infection risk, mitigates symptoms, and shortens the time to recovery.

disease							
Ursodeoxycholic acid is associated with a reduction in SARS-CoV-2 infection and reduced severity of COVID-19 in patients with cirrhosis	John, *et al.* (3)	Cirrhosis	NA	1607	1607	Yes	UDCA exposure was linked to lower odds of contracting SARS-CoV-2 infection and, among COVID-19 patients, reduced disease severity
Ursodeoxycholic Acid Does Not Improve COVID-19 Outcome in Hospitalized Patients	Colapietro, *et al.*(4)	Hospitalized Patients	NA	57	3790	No	Treatment with UDCA did not independently predict survival in hospitalized COVID-19 patients.
Ursodeoxycholic acid administration did not reduce susceptibility to SARS-CoV-2 infection in children	Liu, *et al.*(5)	Children	NA	146	80	No	SARS-CoV-2 infection rates were similar in children taking and not taking UDCA, with no significant differences.

Reference:

1. Brevini T, Maes M, Webb GJ, John BV, Fuchs CD, Buescher G, et al. FXR inhibition may protect from SARS-CoV-2 infection by reducing ACE2. Nature. 2023;615(7950):134-42.
2. Li Y, Zhu N, Cui X, Lin Y, Li X. Protective effect of ursodeoxycholic acid on COVID-19 in patients with chronic liver disease. Front Cell Infect Microbiol. 2023;13:1178590.
3. John BV, Bastaich D, Webb G, Brevini T, Moon A, Ferreira RD, et al. Ursodeoxycholic acid is associated with a reduction in SARS-CoV-2 infection and reduced severity of COVID-19 in patients with cirrhosis. J Intern Med. 2023;293(5):636-47.
4. Colapietro F, Angelotti G, Masetti C, Shiffer D, Pugliese N, De Nicola S, et al. Ursodeoxycholic Acid Does Not Improve COVID-19 Outcome in Hospitalized Patients. Viruses. 2023;15(8).
5. Liu T, Wang JS. Ursodeoxycholic acid administration did not reduce susceptibility to SARS-CoV-2 infection in children. Liver Int. 2023;43(9):1950-4.
